# The Effect of Iatrogenic Hypothyroidism on Lipoprotein Subfractions and Markers of HDL Function in Patients with Differentiated Thyroid Carcinoma

**DOI:** 10.3390/life16071083

**Published:** 2026-06-28

**Authors:** Mónika Katkó, Annamária Gazdag, Anita Szentpéteri, Hajnalka Lőrincz, Erika Galgóczi, Annamária Erdei, Eszter Berta, Miklós Bodor, Endre V. Nagy, Mariann Harangi

**Affiliations:** 1 Division of Endocrinology, Department of Internal Medicine, Faculty of Medicine, University of Debrecen, 4032 Debrecen, Hungary; katko.monika@med.unideb.hu (M.K.);; 2 Division of Metabolism, Department of Internal Medicine, Faculty of Medicine, University of Debrecen, 4032 Debrecen, Hungary; 3 Department of Clinical Basics, Faculty of Pharmacy, University of Debrecen, 4032 Debrecen, Hungary; 4 Doctoral School of Health Sciences, University of Debrecen, 4032 Debrecen, Hungary

**Keywords:** hypothyroidism, lipoprotein subfraction, paraoxonase-1, LDL size, HDL function, thyroid cancer, personalized medicine

## Abstract

**Background/Objectives**: We aimed to conduct a comprehensive assessment of how transient iatrogenic hypothyroidism, induced for diagnostic purposes during the follow up of patients with differentiated thyroid cancer, impacts both quantitative and qualitative lipid parameters. **Methods**: Blood samples were collected during continuous levothyroxine (LT4) supplementation and after four weeks of LT4 withdrawal. In addition to thyroid hormone levels and routine lipid parameters, LDL and HDL subfractions were analyzed using polyacrylamide gel electrophoresis (Lipoprint). Furthermore, the activities of HDL-associated human paraoxonase-1 (PON1) paraoxonase and arylesterase were measured spectrophotometrically, while the levels of myeloperoxidase and apolipoprotein M (ApoM) were determined using ELISA. The activity of key regulators in HDL remodeling was measured using activity assay kits. **Results**: In this prospective, single-center study, a total of 52 patients were enrolled (mean age 48 ± 15 years; 13 males and 39 females). Compared to values measured during continuous LT4 supplementation, total cholesterol, HDL-C, LDL-C, ApoA1, and ApoB100 levels were significantly elevated during iatrogenic hypothyroidism (*p* < 0.0001 for all parameters). Differences in lipoprotein subfraction patterns were also observed: in hypothyroidism, the mean LDL particle size decreased (*p* = 0.0007) and the proportion of HDL subfractions shifted to the larger HDL subfractions (*p* < 0.0001). The paraoxonase activity and ApoM level tended to be increased (*p* = 0.030 and *p* = 0.011, respectively). **Conclusions**: In short-term overt hypothyroidism, opposing changes were observed: the shift toward smaller, denser LDL subfractions is considered atherogenic, whereas the increased proportion of larger HDL subfractions, the trend for higher paraoxonase activity and apoM levels can be potentially anti-atherogenic. Our findings further characterize the functional alterations of lipoproteins in hypothyroidism.

## 1. Introduction

Thyroid function exerts a significant influence on lipid metabolism, affecting the synthesis, mobilization, and degradation of lipoproteins. Previous studies have demonstrated that thyroid hormones increase the activity of several key enzymes involved in lipid metabolism, including 3-hydroxy-3-methylglutaryl coenzyme A, cholesteryl ester transfer protein (CETP), hepatic lipase, and lecithin–cholesterol acyltransferase (LCAT) [1,2]. In addition, they enhance the expression of the low-density lipoprotein (LDL) receptor and ATP-binding cassette transporter A1 [3,4,5]. Although previous studies have predominantly examined alterations in lipid levels in subclinical hypothyroidism [6], data are also available regarding lipid parameters in patients with overt hypothyroidism. These studies demonstrated elevated total cholesterol and LDL-cholesterol (LDL-C) levels, while in some cases, higher triglyceride (TG) and lower high-density lipoprotein-cholesterol (HDL-C) levels were observed; in contrast, other studies reported no significant changes in TG and HDL-C concentrations [7,8,9,10]. Lipoprotein(a) (Lp(a)) levels have likewise been found to be elevated in overt hypothyroidism [11]. In patients who have undergone thyroidectomy and developed acute-onset overt hypothyroidism, similar lipid abnormalities have been observed [12]. In a study involving 27 thyroidectomized patients, in addition to the aforementioned lipid alterations, elevated apolipoprotein B100 (ApoB100) levels and an increased ApoA1/A2 ratio were reported, along with reduced human paraoxonase-1 (PON1) paraoxonase activity and impaired cholesterol efflux [13]. Another study conducted on 18 patients did not observe a significant decrease in CETP and LCAT concentrations or in PON1 activity. No differences were identified in the HDL particle size or lipid composition [14].

Although changes in routinely measured lipid parameters already indicate dyslipidemia and the consequent increased risk of atherosclerosis, the characterization of alterations in lipoprotein subfractions may be of particular importance, as these can help elucidate the pathophysiological background of progressive atherosclerosis observed even in subclinical hypothyroidism [15]. Beyond HDL-C levels, a detailed assessment of HDL function is especially important. This includes measuring PON1 paraoxonase and arylesterase activities, along with other HDL-associated proteins like pro-oxidant myeloperoxidase (MPO) [16] and vasoprotective apolipoprotein M (apoM) [17]. To date, no studies have comprehensively investigated the complex alterations in lipid metabolism associated with overt hypothyroidism.

Therefore, we aimed to investigate patients who had undergone thyroidectomy and ablative radioiodine therapy for differentiated thyroid cancer, first during substitution therapy and subsequently during a diagnostically induced period of acute overt hypothyroidism. We assessed lipid parameters, LDL and HDL subfractions, and HDL functional markers, including PON1 paraoxonase and arylesterase activities, ApoM and MPO concentrations, and the activities of lipid metabolism-related enzymes such as LCAT, CETP, and phospholipid transfer protein (PLTP). We hypothesized that, with the development of overt hypothyroidism, in addition to characteristic changes in lipid levels, unfavorable alterations would occur in the distribution of LDL and HDL subfractions, in HDL functional parameters, and in the activities of enzymes involved in lipid metabolism.

## 2. Materials and Methods

### 2.1. Study Population

Fifty-two patients with differentiated thyroid cancer (mean age: 48 ± 15 years, min-max: 19–78 years), including 13 males and 39 females who underwent total thyroidectomy and subsequent ablative radioiodine therapy, were enrolled in the study. Blood samples were collected during continuous thyroid stimulating hormone (TSH)-suppressive levothyroxine therapy (on-thyroxine) and after four weeks of levothyroxine withdrawal (off-thyroxine). The exclusion criteria included the presence of coronary heart disease, chronic liver or renal disease, and uncontrolled diabetes mellitus, as well as the use of lipid-lowering agents or other treatments that could potentially affect the lipid profile. Detailed information on medication use and prior medical history was obtained for each patient. Data about risk stratification and the target TSH range for each patient were not collected. All participants gave written informed consent. The study was conducted in accordance with the Helsinki Declaration. The study protocol was approved by the Regional Ethics Committee of the University of Debrecen (protocol code: RKEB/IKEB: 4739/2017, date of approval: 20 February 2017).

### 2.2. Blood Sampling

Venous blood samples were obtained after a 12 h overnight fast. Laboratory markers—including TSH, free thyroxine (fT4), free triiodothyronine (fT3), high-sensitivity C-reactive protein (hsCRP), total cholesterol, TG, HDL-C, LDL-C, ApoA1, ApoB100, and Lp(a)—were measured from fresh serum using Cobas c600 analytical systems (Roche Ltd., Mannheim, Germany). All assays were conducted from same vendor according to the respective manufacturers’ instructions. Laboratory analyses were performed at the Department of Laboratory Medicine, University of Debrecen. Non-HDL-C is calculated by subtracting HDL-C from total cholesterol. For ELISA-based determinations, five aliquots of serum (each approximately 300–400 μL) were stored in Eppendorf tubes at −80 °C until analysis.

### 2.3. Measurement of ApoM

Serum ApoM levels were determined with an ELISA kit (BioVendor—Laboratorni medicina a.s., Brno, Czech Republic) and expressed as µg/mL. Intra-assay coefficients of variation ranged from 4.9 to 5.22% and inter-assay coefficients of variation ranged from 5.7 to 5.8%.

### 2.4. Myeloperoxidase Measurement

Serum concentrations of MPO were measured via ELISA (R&D Systems Europe Ltd., Abington, UK), and the assay was performed according to the instructions of the manufacturer. Intra- and inter-assay coefficients of variation were 6.5 and 9.4%, respectively.

### 2.5. Determination of PON1 Enzyme Activities

Serum PON1 paraoxonase activity was monitored using a kinetic, semiautomated method using paraoxon (O,O-diethyl-O-p-nitrophenyl phosphate, Sigma-Aldrich, St. Louis, MO, USA) as a substrate. The hydrolysis of paraoxon was followed at 405 nm at +22–24 °C. Serum PON1 arylesterase activity was measured using phenylacetate as a substrate (Sigma-Aldrich, St. Louis, MO, USA), and the hydrolysis of the substrate was monitored at 270 nm at +22–24 °C, as previously described [18].

### 2.6. Determination of CETP, LCAT, and PLTP Activities

Serum CETP, LCAT, and PLTP activities were determined with commercially available activity assay kits (Cat. Nos. MAK106-1KT, MAK107-1KT, MAK108-1KT, respectively; Sigma-Aldrich, St. Louis, MO, USA) according to the instructions of the manufacturer. Briefly, the CETP Activity Assay Kit uses a proprietary substrate that enables the detection of the CETP-mediated transfer of neutral lipid from the substrate to a physiological acceptor. The transfer activity results in an increase in fluorescence intensity. Data from CETP measurements are presented as µmol/h/mL. The LCAT enzyme activity was evaluated as a ratio change of the fluorescence intensity of the substrate at 390 to 470 nm, and presented as %. The PLTP Activity Assay Kit includes proprietary substrates to detect the PLTP-mediated transfer of the fluorescent substrate. Transfer activity results in increased fluorescent emission intensity (λEx = 465 nm/λEm = 535 nm) from the assay. Data are presented as nmol/h/mL.

### 2.7. Determinations of Lipoprotein Subfractions

LDL and HDL lipoprotein subfractions were distributed based on their size using the Lipoprint System (Quantimetrix Corporation, Redondo Beach, CA, USA), as previously described [19]. Briefly, 25 µL of sera was taken into polyacrylamide gel tubes with 200 and 300 µL of loading gel containing Sudan Black, respectively. After 30 min of photopolymerization, the gel tubes were electrophorized in an electrophoresis chamber with 3 mA/each tube. Each electrophoresis was loaded with a high-purity lipoprotein quality control, which was provided by Quantimetrix (Liposure Serum Lipoprotein Control, Quantimetrix Corp., Redondo Beach, CA, USA). After a half hour but no longer than 2 h of rest, the lipoprotein bands were scanned with an ArtixScan M1 digital scanner (Microtek International Inc., Hsinchu, Taiwan) and analyzed with the Lipoware Image SXM v.1.82 Software developed by the manufacturer (Quantimetrix Corp., Redondo Beach, CA, USA).

In the case of the LDL subfraction analysis, up to seven LDL subfractions were determined between the very low-density lipoprotein (VLDL) and HDL peaks. The proportion of intermediate density lipoprotein (IDL) was defined as the sum of midbands C through A; the proportion of large LDL (large LDL %) was defined as the summed percentages of LDL1 and LDL2, whereas the proportion of the small LDL (small-dense LDL %) was defined as the sum of LDL3–LDL7. Cholesterol concentrations of the LDL subfractions were determined by multiplying the relative area under the curve (AUC) of subfractions by the total cholesterol concentration.

In the case of the HDL subfraction analysis, 10 HDL subfractions were determined: large (HDL1–3), intermediate (HDL4–7), and small (HDL8–10) HDL subfractions were distributed between the VLDL + LDL and albumin bands. The cholesterol content of the HDL subfractions was calculated using Lipoware Software according to the relative AUC of subfraction bands.

### 2.8. Statistical Analyses

Statistical analyses were performed using Statistica 13.5.0.17 (TIBCO Software Inc., Tulsa, OK, USA), and graphs were made using GraphPad Prism 6.01 (GraphPad Prism Software Inc., San Diego, CA, USA). The normality of the continuous variables was tested using the Kolmogorov–Smirnov test. The comparison between groups was analyzed using the Student’s paired t-test or Wilcoxon matched pairs test, respectively. Data were presented as means ± standard deviations or medians (upper and lower quartiles). The relationship between variables was assessed with Pearson tests. Bonferroni correction was applied, and *p* < 0.0015 was considered statistically significant.

## 3. Results

Routine laboratory parameters are presented in Table 1. During the on-thyroxine period, TSH levels were suppressed, in accordance with therapeutic guidelines, in 34 patients, while 18 patients were within the normal range. As expected, significantly higher TSH levels were observed during the off-thyroxine period. fT3 and fT4 levels were significantly lower in the off-thyroxine period compared with the on-thyroxine values. Regarding lipid parameters, total cholesterol, non-HDL-C, LDL-C, TG, and ApoB100 levels were, as anticipated, significantly higher in the off-thyroxine samples compared to the on-thyroxine samples. Interestingly, HDL-C and ApoA1 levels were also found to be significantly higher during the off-thyroxine period. hsCRP and Lp(a) levels did not differ between the on-thyroxine and off-thyroxine periods (Table 1).

**Figure 1:**
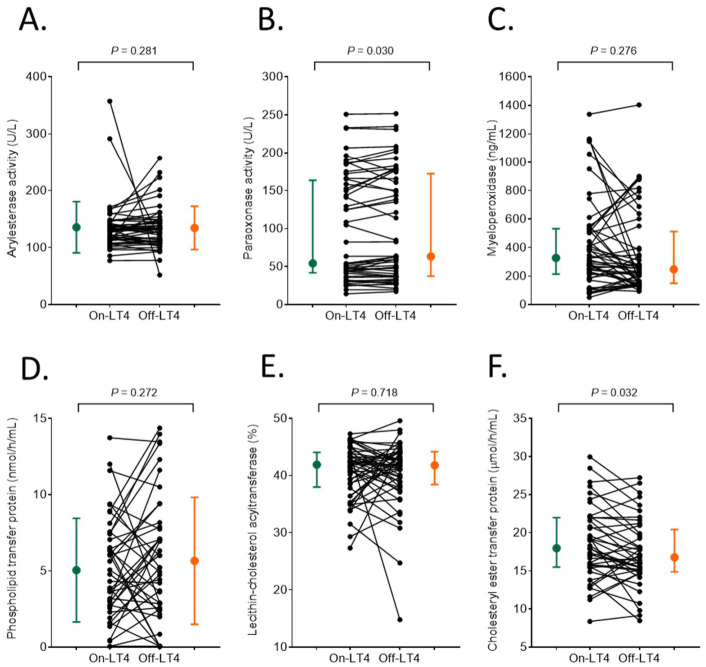
Individual changes (black) and medians of (**A**) arylesterase activity (U/L), (**B**) paraoxonase activity (U/L), (**C**) myeloperoxidase level (ng/mL), (**D**) phospholipid transfer protein activity (nmol/h/mL), (**E**) lecithin–cholesterol acyltransferase activity (%), and (**F**) cholesteryl ester transfer protein activity (µmol/h/mL) in patients with differentiated thyroid carcinoma receiving levothyroxine therapy (on-LT4; green) and after four weeks of levothyroxine withdrawal (off-LT4, orange).

Among the parameters characterizing HDL function, the antioxidant activities of PON1 paraoxonase tended to be higher during the off-thyroxine period, although both activities showed considerable interindividual variability (Table 2). The pro-oxidant MPO level did not differ between the two periods. The level of the HDL-associated protein ApoM showed an upward trend during the off-thyroxine period. CETP activity was higher in most of the patients in the on-thyroxine period compared with the off-thyroxine values, but the difference did not reach statistical significance after Bonferroni correction, whereas no differences were observed in the activities of LCAT and PLTP (Figure 1A–F).

**Figure 2:**
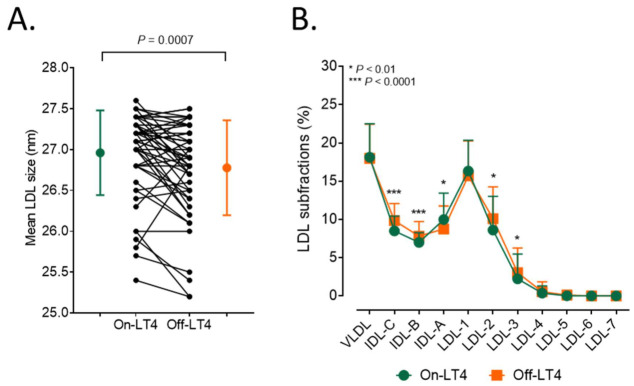
(**A**) Individual changes (black) and medians of mean low-density lipoprotein (LDL) particle size and (**B**) the proportion of LDL subfractions in patients with differentiated thyroid carcinoma receiving levothyroxine therapy (on-LT4; green) and after four weeks of levothyroxine withdrawal (off-LT4, orange).

**Figure 3:**
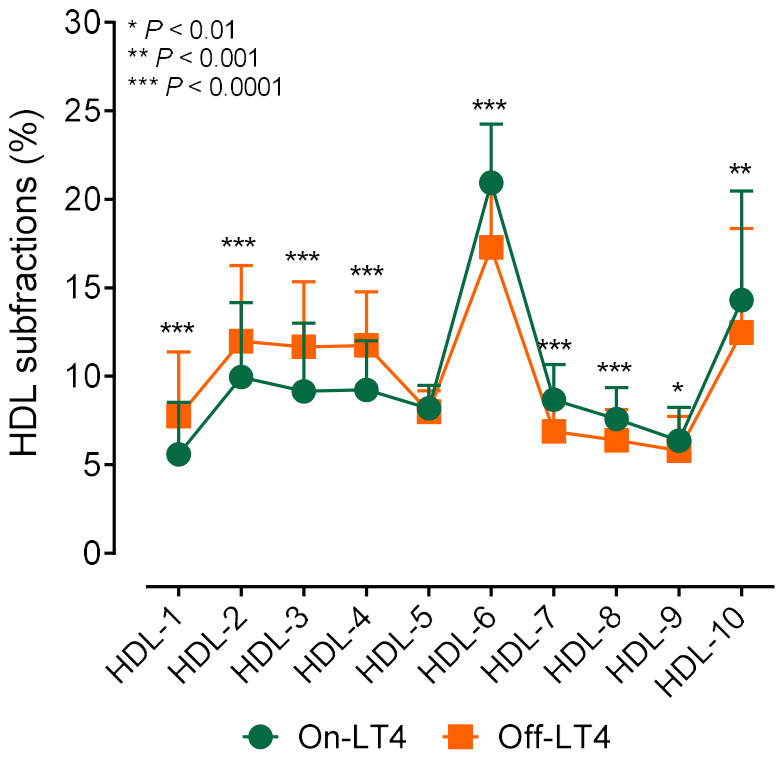
Proportion of high-density lipoprotein (HDL) subfractions in patients with differentiated thyroid carcinoma receiving levothyroxine therapy (on-LT4; green) and after four weeks of levothyroxine withdrawal (off-LT4, orange).

The mean LDL particle size was significantly smaller during the off-thyroxine period (Figure 2A and Table 2). An analysis of LDL subfraction portions revealed a shift toward smaller particles in the off-thyroxine period (Figure 2B and Table 2). Furthermore, the portion of HDL subfractions shifted toward larger particles during the off-thyroxine period (Figure 3). The absolute amounts of VLDL, IDL, large LDL, small-dense LDL, large HDL, and intermediate HDL subfractions were found to be significantly higher in the off-thyroxine period compared with the on-thyroxine period (Table 2).

In the on-thyroxine period, fT4 showed a positive correlation with fT3 (r = 0.310; *p* = 0.025), whereas there were negative correlations among TG, ApoM, and fT4 levels, respectively (r = −0.297; *p* = 0.033 and r = −0.351; *p* = 0.011). The levels of fT3 showed negative correlations with total cholesterol (r = −0.386; *p* = 0.005), non-HDL-C (r = −0.300; *p* = 0.031), LDL-C (r = −0.325; *p* = 0.019), and ApoA1 (r = −0.359; *p* = 0.009) in the on-thyroxine period.

## 4. Discussion

To the best of our knowledge, considering both the specific combination of evaluated parameters and the methodologies applied, this is the only study to comprehensively assess routine lipid parameters, lipoprotein subfractions, HDL function, and enzyme concentrations related to lipid metabolism in patients with differentiated thyroid carcinoma receiving continuous levothyroxine therapy and after four weeks of levothyroxine withdrawal.

Although most lipid alterations are in agreement with previous reports [20], in our study population, overt hypothyroidism developing during the off-thyroxine period was unexpectedly associated with increased HDL-C levels. This may be partly explained by an increased CETP activity in most of the patients at the on-thyroxin period, as CETP mediates the transfer of triglycerides from other lipoproteins, such as LDL and VLDL particles, to HDL, while concurrently transferring cholesteryl esters from HDL back to these particles. This process promotes HDL maturation and an increase in particle size, which may account for the observed shift in the proportion of HDL subfractions toward larger HDL particles. However, these findings are not consistent with those of a previous study, albeit conducted in a smaller cohort, in which no difference in CETP levels was observed in patients with overt hypothyroidism following thyroidectomy [14]. Although hepatic lipase activity was not measured in the present study, previous human studies in subclinical hypothyroidism, as well as experimental animal models of hypothyroidism, have reported a reduction in HDL activity [21,22]. Hepatic lipase acts as a negative regulator of HDL levels by hydrolyzing triglycerides and phospholipids within the particles. Consequently, low hepatic lipase activity results in higher plasma HDL-C levels [23]. Therefore, reduced hepatic lipase activity may contribute to the higher HDL-C levels observed in our study population.

Data on LDL and HDL subfractions in hypothyroidism are limited, with previous findings derived primarily from studies involving patients with subclinical hypothyroidism. Another study did not find significant changes in the HDL particle size and lipid composition in patients after thyroidectomy with overt hypothyroidism [14]. According to the results of the ELSA-Brasil study, subclinical hypothyroidism is characterized by a significantly lower proportion of small HDL subfractions and a higher proportion of intermediate HDL subfractions [24]. A previous study did not find significant alterations in HDL subfractions, while there was a significant decrease in the LDL size and higher prevalence of atherogenic pattern B [25]. A further study using isopycnic ultracentrifugation in patients with primary hypothyroidism showed that the HDL subclass distribution is altered under hypothyroid conditions. Specifically, both the less dense HDL fraction (HDL2b) and the denser subclasses (HDL3b+3c) were elevated, whereas the intermediate-density subfraction (HDL2a and 3a) remained unchanged [26]. Our findings are consistent with the majority of the literature, demonstrating that during the off-thyroxine period, the proportion of large HDL subfractions increased. Although several previous studies have demonstrated a negative correlation between HDL particle size and cardiovascular risk [27,28,29,30], recent studies reveal a more nuanced picture where small, dense particles play critical roles in sub clearing excess cholesterol. Indeed, small HDL particles are more effective than large HDL particles at inducing cholesterol efflux from cells via ABCA1. In contrast, large HDL particles are more effective at preventing LDL transcytosis through endothelial cells [31]. It should be emphasized that a wide range of clinical conditions have been linked to distinct HDL subfractions; however, substantial research is still required to establish causal relationships and to reconcile inconsistent findings arising from the use of different separation techniques and classification methods [32].

According to the literature, HDL function is a more important determinant of cardiovascular risk than HDL-C levels per se, and this function is largely mediated by HDL-associated enzymes [33]. The antiatherogenic properties of PON1, particularly its antioxidant effects, have been extensively studied and demonstrated in numerous investigations [34]. The antioxidant capacity is reflected by the enzyme’s paraoxonase activity, whereas the quantity of the PON1 protein is characterized by its arylesterase activity [35]. Reduced paraoxonase activity has been demonstrated in several patient populations with an increased risk of atherosclerosis [36,37,38,39]. However, in patients with hypothyroidism, most previous studies have not observed significant changes in PON1 activity [14,40]. In one study, paraoxonase activity normalized to ApoA1 was found to be decreased [13]. Based on our results, although arylesterase activity remained unchanged, paraoxonase activity tended to be higher during the off-thyroxine period. On the surface of HDL, PON1 forms a complex with ApoA1 and the pro-oxidant enzyme MPO, and PON1 and MPO mutually inhibit each other’s activity [41]. MPO levels, although not significantly, were lower during the withdrawal period, which may explain the higher paraoxonase activity.

ApoM is a 26 kDa protein that belongs to the lipocalin family and is predominantly associated with HDL particles that coordinate a range of vasoprotective effects, primarily through the transport of sphingosine-1-phosphate [17]. It plays an important role in maintaining endothelial barrier integrity and in regulating nitric oxide production, vascular tone, and vascular inflammation [42,43]. In addition, circulating HDL-associated ApoM is critically involved in transendothelial HDL transport and cholesterol efflux [44]. Previous studies have demonstrated an association between reduced ApoM expression and the development of cardiometabolic diseases; however, there is also growing evidence supporting a role for ApoM in neurovascular, inflammatory, and retinal disorders [42]. ApoM expression is mainly regulated by hepatocyte nuclear factors, Forkhead box O transcription factors, and inflammatory cytokines [45]. Furthermore, its production can be modulated pharmacologically by certain drugs, including statins and SGLT2 inhibitors [46,47]. To date, the effect of hypothyroidism on the ApoM concentration has not been investigated. The trend towards an elevated ApoM concentration observed during the off-thyroxine period represents a novel finding and may indicate increased expression induced by hypothyroidism, potentially reflecting an adaptive response to the hypothyroid state.

Triglyceride-rich lipoprotein (TRL) particles are increasingly recognized as important contributors to atherogenesis [48]. Smaller particles can infiltrate the subendothelial space and, owing to their size and extended retention, facilitate foam cell formation and trigger local inflammatory processes [49]. A prior study showed that severe hypothyroidism following thyroidectomy leads to marked elevations in circulating TRL and LDL particle concentrations, with the most pronounced increase observed in very small TRL (remnant) particles [50]. In line with these previous data, we also found increased levels of TRL particles (IDL and VLDL) during the off-thyroxine period.

In a systematic review and meta-analysis using data from 1970 to 2018 levothyroxine therapy in overt hypothyroidism showed a statistically significant decrease in total cholesterol, LDL-C, HDL-C, triglycerides, apoA, apoB, and Lp(a) [51]. These variations align with our findings, showing that levothyroxine treatment produces the exact opposite effects of treatment withdrawal, with Lp(a) being the sole exception to this trend. It is demonstrated that overt hypothyreoidism leads to elevated circulating levels of lipoprotein (a) and starting LT4 replacement therapy significantly reduces it [11]. However, no alteration in Lp(a) was found after 4-week LT4 withdrawal in our study.

The findings of this study may also have implications for patient management. Although the discontinuation of levothyroxine is short-term, it is nevertheless associated with the development of substantial hypercholesterolemia and hypertriglyceridemia, which, based on the cumulative LDL hypothesis, may still result in an increased cardiovascular risk [52]. However, our data are primarily relevant to understanding the pathophysiology of hypothyroidism-induced dyslipidemia and do not support the need for routine lipid-lowering therapy during short-term diagnostic LT4 withdrawal unless it is clinically indicated. Given that cardiovascular outcomes were not evaluated in this study, it remains unclear whether transient alterations in lipid metabolism elevate risk within this cohort. Notably, in patients with differentiated thyroid cancer, both sustained subclinical hyperthyroidism and acute diagnostic hypothyroidism represent potential contributors to overall cardiovascular risk.

The strength of our study lies in its methodological breadth, enabling a comprehensive, simultaneous evaluation of lipid metabolism in this specific patient population. Nevertheless, several limitations should be considered. Although our sample size is comparable to previous studies, it is still modest for extensive subgroup analyses, which was avoided to prevent underpowered statistical comparisons. Furthermore, the substantial inter-individual variability in some parameters may have reduced statistical power. Consequently, these results should be interpreted with strict caution and viewed as exploratory findings. Despite efforts to exclude comorbidities and treatments that could confound the results, environmental or dietary factors during the two study periods may have contributed to observed differences, rather than changes in thyroid hormone levels alone. Specifically, potential confounding factors such as short-term dietary changes, weight fluctuations, and variations in physical activity during the LT4 withdrawal period were not strictly monitored. However, given the short duration of the withdrawal phase, shifts in body composition or lifestyle habits were unlikely to fully account for the substantial metabolic and physiological changes observed. Additionally, variations in ethnicity, age, and other patient characteristics including THS levels during LT4 supplementation may have influenced our findings and could explain discrepancies with prior reports. Moreover, the alterations observed by us after short-term hypothyroidism may differ from those associated with long-term, untreated disease.

## 5. Conclusions

In addition to the observed alterations in serum lipid levels during short-term iatrogenic hypothyroidism, the subfractional pattern of lipoproteins also changes. The deviations observed in the subfractional pattern suggest a pro-atherogenic effect for LDL, whereas for HDL, they might indicate predominantly anti-atherogenic effects. Further studies are needed to elucidate the precise mechanisms underlying these changes and to determine whether the changes observed in our study persist in milder and/or long-term hypothyroidism.

## Figures and Tables

**Table 1:** The effect of 4-week levothyroxine withdrawal on routine laboratory parameters in patients with differentiated thyroid carcinoma.

Variable	On-Thyroxine	Off-Thyroxine	*p*-Value
TSH (mU/L)	0.094 (0.023–0.556) <0.005–3.520	98.565 (75.895–>100.000) 47.950–>100.000	<0.0001
fT4 (pmol/L)	23.7 (21.9–26.7) 14.5–34.4	2.1 (1.5–2.9) 0.9–4.9	<0.0001
fT3 (pmol/L)	5.0 (4.7–5.6) 3.3–6.5	1.3 (0.9–1.5) 0.4–2.1	<0.0001
hsCRP (mg/L)	1.8 (0.7–4.2) <0.5–14.8	1.5 (<0.5–4.1) <0.5–15.5	0.198
Triglyceride (mmol/L)	1.5 (0.9–2.0) 0.4–5.7	2.0 (1.4–2.8) 0.4–5.5	<0.0001
Total cholesterol (mmol/L)	5.0 (4.4–6.2) 3.3–8.7	7.3 (6.3–8.8) 4.0–10.8	<0.0001
HDL-C (mmol/L)	1.4 (1.1–1.6) 0.7–2.1	1.8 (1.4–2.1) 0.9–2.7	<0.0001
LDL-C (mmol/L)	3.1 (2.3–4.0) 1.7–6.5	4.9 (3.9–6.1) 2.1–8.7	<0.0001
nonHDL-C (mmol/L)	3.6 (2.9–5.0) 1.8–7.5	5.5 (4.6–6.8) 2.1–9.9	<0.0001
Apolipoprotein A1 (g/L)	1.59 (1.44–1.77) 1.06–2.14	1.89 (1.66–2.12) 1.11–2.53	<0.0001
Apolipoprotein B100 (g/L)	0.97 (0.78–1.26) 0.59–1.83	1.42 (1.14–1.72) 0.7–2.4	<0.0001
Lipoprotein (a) (mg/L)	32 (<30–204) <30–1436	33 (<30–180) <30–1501	0.626

Abbreviations: fT4, free thyroxine; fT3, free triiodothyronine; HDL-C, high-density lipoprotein cholesterol; hsCRP, high sensitivity C-reactive protein; LDL-C, low-density lipoprotein cholesterol; TSH, thyroid stimulating hormone. Data are presented as the median (interquartile ranges) and minimum–maximum values. *p*-values were calculated using Wilcoxon matched pairs test.

**Table 2:** The effect of 4-week discontinued levothyroxine on markers of HDL function and lipoprotein subfractions.

Variable	On-Thyroxine	Off-Thyroxine	*p*-Value
Markers of HDL function			
PON1 paraoxonase activity (U/L)	54.2 (42.1–161.8) 14.1–250.8	63.3 (37.7–170.0) 17.1–251.7	0.030
PON1 arylesterase activity (U/L)	129.6 (115.1–142.5) 77.2–357.3	131.6 (108.9–148.2) 51.2–257.4	0.281
Myeloperoxidase (ng/mL)	328 (217–532) 51–1338	248 (151–474) 93–1403	0.276
Apolipoprotein M (µg/mL)	3.05 (2.79–3.36) 2.27–4.52	3.20 (2.92–3.60) 2.15–4.53	0.011
PLTP activity (nmol/h/mL)	4.77 (2.56–7.41) 0.05–13.73	5.20 (2.48–7.88) 0.05–14.37	0.272
CETP activity (µmol/h/mL)	17.97 (15.53–21.95) 8.37–29.95	16.76 (15.04–19.88) 8.47–27.21	0.032
LCAT activity (%)	41.9 (38.2–44.0) 27.3–53.0	41.8 (38.6–44.2) 14.8–51.2	0.718
Portion of lipoprotein subfractions			
Mean LDL particle size (nm)	27.1 (26.8–27.3) 25.4–27.6	26.9 (26.5–27.2) 25.2–27.5	0.0007
VLDL (%)	18.5 (14.6–21.3) 10.9–28.7	18.7 (14.9–21.6) 9.7–27.9	0.659
IDL (1–3) (%)	25.1 (22.1–29.1) 14.3–36.0	26.4 (24.2–29.0) 16.9–42.1	0.232
Large LDL (1–2) (%)	24.7 (22.75–27.90) 13.8–35.9	26.8 (23.1–29.3) 12.6–36.0	0.181
Small-dense LDL (3–7) (%)	1.4 (0.0–2.7) 0.0–16.6	1.7 (0.5–4.9) 0.0–17.7	0.007
Large HDL (1–3) (%)	24.7 ± 9.9 7.2–43.7	31.4 ± 10.4 12.3–53.4	<0.0001
Intermediate HDL (4–7) (%)	47.0 ± 4.8 37.3–54.7	43.9 ± 4.7 35.6–53.2	<0.0001
Small HDL (8–10) (%)	28.3 ± 9.3 15.1–53.8	24.6 ± 9.0 9.9–47.0	<0.0001
Amount of lipoprotein subfractions			
VLDL (mmol/L)	0.92 (0.67–1.18) 0.43–1.97	1.37 (0.97–1.72) 0.40–2.47	<0.0001
IDL (1–3) (mmol/L)	1.27 (1.02–1.67) 0.64–2.65	1.98 (1.49–2.37) 0.72–3.71	<0.0001
Large LDL (1–2) (mmol/L)	1.29 (0.97–1.60) 0.48–2.63	1.98 (1.47–2.21) 0.75–3.41	<0.0001
Small-dense LDL (3–7) (mmol/L)	0.06 (0.00–0.18) 0.00–1.04	0.12 (0.03–0.43) 0.00–1.56	<0.0001
Large HDL (1–3) (mmol/L)	0.37 ± 0.20 0.072–0.813	0.59 ± 0.32 0.122–1.388	<0.0001
Intermediate HDL (4–7) (mmol/L)	0.66 ± 0.17 0.349–0.970	0.78 ± 0.20 0.365–1.188	<0.0001
Small HDL (8–10) (mmol/L)	0.38 ± 0.10 0.204–0.607	0.41 ± 0.12 0.190–0.799	0.006

Abbreviations: CETP, cholesteryl ester transfer protein; IDL, intermediate density lipoprotein; HDL, high-density lipoprotein; LCAT, lecithin–cholesterol acyltransferase; LDL, low-density lipoprotein; PLTP, phospholipid transfer protein; PON1, human paraoxonase-1. Data are presented as the median (interquartile ranges) or mean ± standard deviation with minimum–maximum values. *p*-values were calculated using Wilcoxon matched pairs or Student’s paired tests.

## Data Availability

The original contributions presented in this study are included in the article. Further inquiries can be directed to the corresponding author.

## Abbreviations

The following abbreviations are used in this manuscript:

ApoB100	apolipoprotein B100
ApoM	apolipoprotein M
AUC	area under the curve
CETP	cholesteryl ester transfer protein
fT3	free triiodothyronine
fT4	free thyroxine
HDL	high-density lipoprotein-cholesterol
HDL-C	HDL-cholesterol
hsCRP	high-sensitivity C-reactive protein
IDL	intermediate density lipoprotein
LCAT	lecithin–cholesterol acyltransferase
LDL	low-density lipoprotein
LDL-C	LDL-cholesterol
Lp(a)	lipoprotein(a)
MPO	myeloperoxidase
PLTP	phospholipid transfer protein
PON1	human paraoxonase-1
TG	triglyceride
TRL	triglyceride-rich lipoprotein
TSH	thyroid stimulating hormone
VLDL	very low-density lipoprotein
